# The Effect of Try-in Paste Shade and Framework Design on the Color Characteristics of Dental Zirconia Restorations 

**DOI:** 10.30476/dentjods.2024.100644.2235

**Published:** 2025-06-01

**Authors:** Abolghasem Mohammadi, Alireza Gerami, Sara Tavakolizadeh, Rahab Ghoveizi, Ehsan Rouhollahpour Ahangar

**Affiliations:** 1 Dept. of Prosthodontics, School of Dentistry, Shahid Beheshti University of Medical Sciences, Tehran, Iran.; 2 Graduate Student, School of Dentistry, Shahid Beheshti University of Medical Sciences, Tehran, Iran.; 3 Postgraduate Student Dept. of Prosthodontics, School of Dentistry, Shahid Beheshti University of Medical Sciences, Tehran, Iran.

**Keywords:** Computer-Aided Design, Crowns, Dental Prosthesis Design, Dental Cements, Zirconia, Try-in paste

## Abstract

**Background:**

Favorable esthetic and mechanical qualities, together with rapid advancements in CAD/CAM technology, have increased the adoption of zirconia restorations. Despite acceptable optical characteristics of zirconia, achieving natural tooth color resemblance remains challenging.

**Purpose:**

This study was conducted to determine how the try-in paste shade, tooth region, and framework design affect the color properties of zirconia restorations.

**Materials and Method:**

In this in vitro study, a maxillary central incisor was prepared and scanned. Frameworks with four different designs were fabricated. Ten crowns were designed in each group categorized as simple core (SC), dentin core (DC), trestle design core (TC), and monolithic crown (MC). Veneering was performed for all groups except MC. Subsequently, all crowns were cemented with try-in paste Bisco CHOICE 2 cement in shades A1-A3 and B1. The color data (Lab) were determined using the SpectroShade Micro II device. Color difference (ΔE) with the Lab B2 color sample as the target color was calculated using the CIE ΔE 2000 formula. Data analysis was performed using the repeated measure ANOVA test.

**Results:**

Zirconia core design, tooth region and cement shade, significantly impacted the ΔE and there were interactions among these factors. The highest ΔE was observed when no cement was used, while the lowest ΔE was observed with A2 cement. Among the various framework designs, the lowest and highest ΔEs were observed in MC and, SC frames, respectively (*p* Value < 0.05).

**Conclusion:**

In the light of the findings of current study, minimum ΔE can be achieved with trestle design framework at the middle portion of the tooth. The try-in paste shade also has a considerable impact on the final ΔE value.

## Introduction

The need for fixed prostheses is increasing due to the growing number of older people in society [ 1 ]. Fixed prostheses offer advantages such as better durability and fewer complications [ 2 - 3 ]. The success of fixed prosthetic treatments depends on factors such as esthetics, fracture resistance, and internal fit. Achieving a natural tooth shade in dental restorations is a challenge due to the complexity of the shade characteristics of natural teeth [ 4 ]. Several factors influence the color of fixed crowns, including the tooth substrate [ 5 ], the cement [ 6 ], the zirconia core [ 7 ], porcelain veneer [ 8 ], and the glaze [ 9 ]. The framework design also plays a role in the final shade of the restoration [ 10 ]. Although metal-ceramic systems are standard [ 11 ], they cannot achieve a natural appearance due to the light-blocking properties of the metal [ 12 - 13 ]. 

For this reason, various all-ceramic systems have been developed and evaluated over the last 45 years to solve the esthetic problems of metal-ceramic crowns [ 14 ]. The main drawbacks of first full-ceramic crowns were inadequate marginal fit and poor physical properties. In recent years, the use of zirconia has significantly improved the physical properties of all-ceramic restorations [ 15 ]. In addition, the CAD/CAM systems used for zirconia crowns are so advanced that the marginal fit of these restorations is comparable to that of metal-ceramic crowns [ 16 ]. As already mentioned, the choice of cement plays a decisive role in the final color of the restoration. Zirconia is a semi-transparent material, which means that the color of the underlying cement will influence the final color of the restoration. Fazi *et al*. [ 17 ] found that even a 0.5mm thick porcelain veneer on zirconia crowns is not sufficient to cover the cement shade. A temporary cement (for short-term esthetics and performance) or a permanent cement is usually used to cement the restoration [ 18 ]. For implant-supported zirconia-based restorations, a temporary cement can be used for a longer period of time [ 19 ]. In order to achieve a better esthetic result, it is advisable to carry out a try-in of the restoration before cementing. This allows the dentist and the patient to assess the color changes of the restoration by using try-in pastes that are the same color as the resin cements [ 20 ]. Several studies have investigated the compatibility between try-in systems and cements [ 21 - 22 ]. Generally, it is believed that the use of try-in paste can practically predict the final esthetic results of resin cements. However, some studies have not reported the color match between resin cements and try-in paste, indicating that the dentist should not rely on the try-in paste for the final color assessment [ 20 ]. Despite the significant influence of cement on the final color of all-ceramic restorations, the effect of cement on the color of zirconia-based restorations has been studied only to a limited extent [ 23 - 26 ], leading to an incomplete understanding of its role [ 19 ]. The aim of this study was to investigate the effects of four types of try-in paste resin cements on the color properties of zirconia crowns with four different framework designs (including simple core, dentin core, trestle design core, and monolithic crown) to help clinicians select the optimal try-in paste shade and framework design to achieve optimal esthetics. Our null hypothesis was that the final color of zirconia restorations would not be affected with tooth region, different designs and try-in paste shades. 

## Materials and Method

The present study was conducted at Shahid Beheshti University of Medical Sciences (Ethical code: IR. SBMU.RIBS.REC.1396.533). In this experimental laboratory study, 40 zirconia cores were divided into four groups (N= 10): simple cores without anatomical contour, dentin core contour, trestle core, and monolithic crowns. A human upper right central tooth that was extracted for periodontal reasons was mounted in a resin model (AcroPars, Marlic Medical Industries Co., Iran) with the resin positioned 3 mm more apically than the cementoenamel junction (CEJ). The tooth silicon index was obtained using Speedex Putty silicone (COLTENE, Germany, Berlin). An intra-oral scanner (LMTmag, Optical 3D Scanner, OpenTechnologies SRL, Italy) was used to scan the unprepared tooth for further design of monolithic crowns. The tooth was prepared anatomically with a turbine and a diamond bur up to 1 mm coronal to the CEJ. Tooth preparation was performed under air and water spray. Based on the guidelines of the Kuraray factory and available instructions to prepare all-ceramic crowns [ 27 ], 1.5mm incisal reduction and 1 mm axial wall reduction with a convergence angle of 6 degrees were performed. The finishing line was a heavy chamfer. After initial preparation, all sharp angles were rounded and the spaces were rechecked with the putty index.

**Figure 1:**
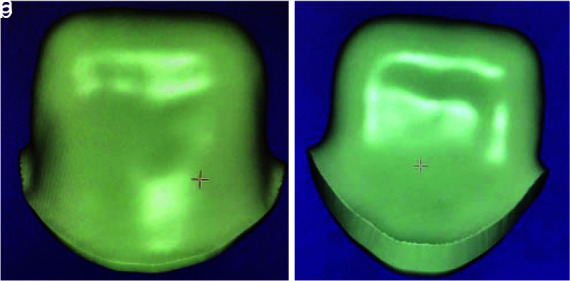
Simple core design, **a:** Buccal view, **b:** Palatal view

**Figure 2:**
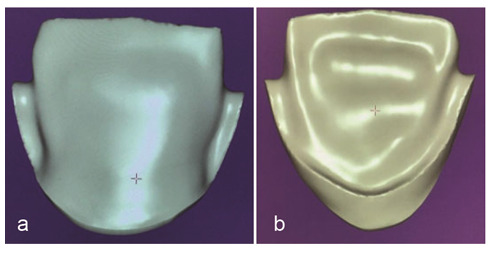
Trestle design, **a:** Buccal view, **b:** Palatal view

**Figure 3:**
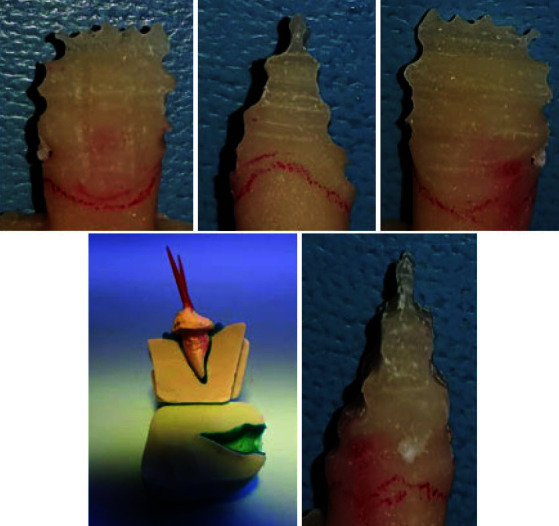
Preparations for dentin core design

**Figure 4:**
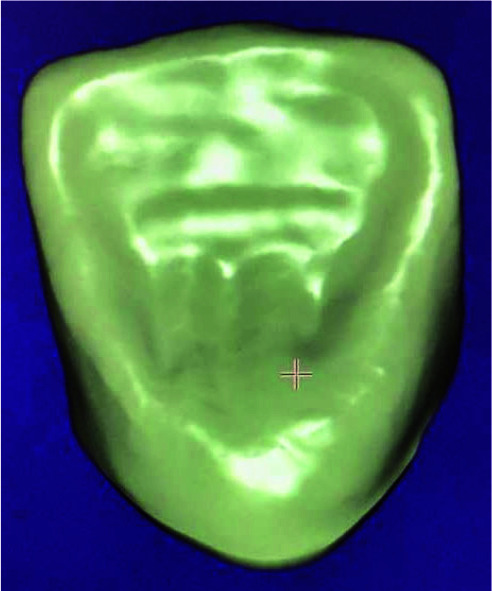
Monolithic design

The IUS3D scanner (Open Technologies Dental, Italy, Brescia) was used to digitally scan the prepared tooth. The frameworks were designed with ExoCAD software (ExoCAD Dental CAD, Darmstadt, Germany). The features of the frameworks were as follows:

- **Simple zirconia core:** Without anatomical contour with a uniform thickness of 0.5 mm (Figure 1).
- **Zirconia core with trestle design contour:** Similar to the porcelain fused to metal (PFM) core with 3 mm collar in the palatal area and proximal buttress up to half the proximal height (Figure 2).

- **Zirconia core with dentin-like contour:** Due to the different thicknesses of dentin and enamel in various tooth portions, it was a challenge to design with ExoCAD. Therefore, an acrylic replica of the tooth was created with an unprepared tooth model. The resin replica was then divided vertically into three sections and horizontally into five sections. Depth cuts with enamel thickness were made in the indicated areas, the depth cuts were connected and the prepared sample was digitally scanned as a dentine core (Figure 3). The core was prepared manually and then scanned to achieve the desired result [ 28 ].

- **Zirconia monolithic crown:** A full contour crown was designed according to the contour of the unprepared tooth (Figure 4).

The ARUM milling machine (Doowon ID Co, Korea) was used to mill the designed frameworks on a KATANA HTML PLUS blank (Kuraray Noritake Dental Inc, Japan). The rationale behind selecting the KATANA HTML PLUS blank was its versatility in being suitable for both framework design and monolithic crowns. Samples without defects were included. In the next step, a trained technician veneered three core groups with feldspar based zirconium oxide veneering ceramic (Zr-FS) (GC, Europe A.G., E.U.) according to the unprepared tooth index. The veneering material consisted of two dentin and enamel powders. First, the framework modifier was heated at 450˚C preheat, followed by dentin at 600˚C preheat and 810˚C. Finally, the glaze process was carried out at 480˚C preheat and 832˚ C. During these processes, the samples and their dimensions were continuously monitored with the index.

After crown preparation, the tooth was placed on a white ionolite plate for color evaluation. The ΔE values were determined in the incisal, middle and cervical tooth regions of the buccal surface. The Panasil white silicone paste was applied to ensure a uniform background and to fix the crown. Shade matching was performed with the SpectroShade Micro II (SpectroShade, USA) according to the Vita Classic criteria and settings for the upper teeth. First, the tooth shade was evaluated with the SpectroShade Micro II, which identified the closest match to B2 and served as a reference for comparison. Bisco CHOICE 2 try-in paste in shades A1, A2, A3, and B1 was used. The color of each crown was examined with the SpectroShade Micro II instrument according to the Vita Classic criteria and specific settings for the upper teeth. Each crown was evaluated five times with the SpectroShade Micro II (once without cement and four times with different cements). After each cement evaluation, the crowns were removed and both the tooth and the crown were rinsed with water. The crowns were then ultrasonically cleaned in distilled water for 2-3 minutes and then dried.

Each analysis included four settings (total color, one-third of the crown, and mapping, for a total of five images). The data included color information based on the Lab CIE system for the entire crown and one-third of each crown. Color changes were measured using the CIE ΔE00 method, the latest technique for evaluating color changes in a sample with a color source identical to that of the tooth and is calculated based on the following formula: 

ΔE_00_[L_1_*, a_1_*, b_1_*; L_2_*, a_2_*, b_2_*] = ΔE^12^_00_ = ΔE_00_ In this formula, (L1*, a1*, b1*) and (L2*, a2*, b2*) are the color values based on the sample Lab and source. This formula can also be adjusted based on the importance of lightness, chroma, and hue.

ΔE generated by SPSS software version 22 (IBM Co., Chicago, IL, USA) were amplified by repeated measure ANOVA. Statistical significance (*p* Value) was determined to be 0.055. 

## Results

**Figure 5:**
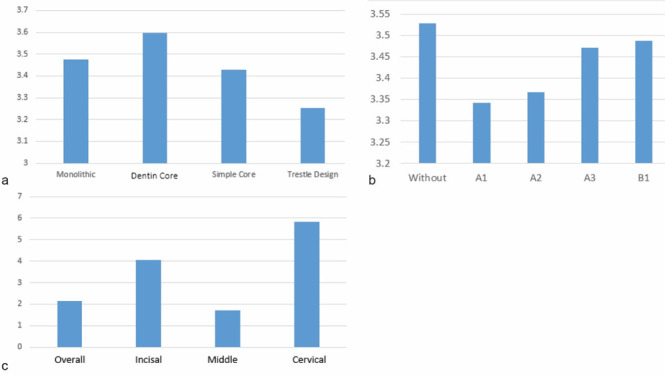
**a:** ΔE based on the type of design, **b:** ΔE based on cement color, **c:** ΔE based on tooth region

The three-way ANOVA test confirmed the significant interaction of the framework design, tooth region and try-in paste shade on ΔE (Table 1). Figures 5 and Tables 2-4 illustrate the average ΔE values in study groups.

**Table 1:** Three-way ANOVA, interaction of the framework design, tooth region and try-in paste shade on ΔE

Source	Type III Sum of Squares^a^	df	Mean Square	F	Sig.^b^
Corrected Model	2396.991^a^	79	30.342	85.659	.000
Intercept	9460.722	1	9460.722	26709.006	.000
Design	12.196	3	4.065	11.477	.000
Location	2163.620	3	721.207	2036.073	.000
Cement	4.165	4	1.041	2.940	.020
Design * Location	168.543	9	18.727	52.869	.000
Design * Cement	9.809	12	.817	2.308	.007
Location * Cement	13.917	12	1.160	3.274	.000
Design * Location * Cement	24.740	36	.687	1.940	.001
Error	255.035	720	.354		
Total	12112.748	800			
Corrected Total	2652.026	799			

^a^ R Squared = .904 (Adjusted R Squared = .893) ^b^ Computed using alpha = .05

**Table 2:** ΔE based on the framework design

Design	Mean	Std. Error	95% Confidence Interval	
			Lower Bound	Upper Bound
Monolithic	3.475	.042	3.393	3.558
Dentin Core	3.597	.042	3.514	3.680
Simple Core	3.430	.042	3.348	3.513
Trestle Design	3.253	.042	3.170	3.336

**Table 3:** ΔE based on try in-paste shade

Cement	Mean	Std. Error	95% Confidence Interval	
			Lower Bound	Upper Bound
Without Cement	3.528	.047	3.436	3.621
A1	3.341	.047	3.248	3.433
A2	3.367	.047	3.275	3.460
A3	3.471	.047	3.379	3.564
B1	3.487	.047	3.394	3.579

**Table 4:** ΔE based on tooth region

Region	Mean	Std. Error	95% Confidence Interval	
			Lower Bound	Upper Bound
Overall	2.158	.042	2.076	2.241
Incisal	4.050	.042	3.968	4.133
Middle	1.702	.042	1.620	1.785
Cervical	5.845	.042	5.762	5.927

The mean ΔE values for each framework design were as follows: monolithic group 2.33±0.300, dentin core group 2.59±0.174, simple core group 2.08±0.333 and trestle design group 2.57±0.207. All framework designs had clinically acceptable ΔE values (< 5.5). 

Comparing the tooth regions, cervical third showed the highest ΔE values (*p*< 0.05). The mean control ΔE values in the incisal region were 2.28±0.321, 5.23± 0.204, 4.04±0.168 and 3.62±1.738, for the monolithic, dentin core, simple core, and trestle design groups, respectively. The results of the ANOVA test showed significant differences between groups (*p*< 0.001).

The mean control ΔE in the middle third for the groups were 1.95±0.297, 2.135±0.234, 1.48±0.364 and 2.09±0.201, respectively. The ANOVA test showed significant differences between groups (*p*< 0.001) and within groups (*p*= 0.001).

In the cervical third, the mean ΔE for the groups were 6.72±0.336, 5.53±0.161, 6.32±0.240 and 5.48± 1.032. The ANOVA test revealed significant differences between the groups (*p*< 0.001).

Considering the try in-paste shade, the highest ΔE was in shade B1 and the lowest ΔE value was observed in A2 (*p*< 0.05). For the monolithic design, the highest ΔE was found in shade A1 and the lowest in shade A2. For the dentin core group, the highest ΔE was in shade B1 and the lowest in A3. In the simple core group, the highest ΔE value was in shade B1 and the lowest in shade A1. For the trestle design, the highest ΔE value was in color B1 and the lowest in A1. Statistical analysis revealed a significant difference in ΔE between the different colors (*p*< 0.001). 

## Discussion

In modern dentistry, advances in dental materials and tools have raised society's expectations for a more natural color, shape, and durability of crowns. The introduction of CAD/CAM technology in dentistry has made the fabrication of all-ceramic crowns almost standard practice [ 29 ]. This technology allows for precise control of the crown manufacturing process and offers a wide range of treatment options. Attaining the intended color by indirect bonded restoration is a crucial prerequisite for achieving a favorable outcome in esthetic rehabilitation procedures [ 29 ]. Based on the findings of our study, the final color of a zirconia restoration is affected by the core design, try in-paste shade, and tooth region. These items also had significant interactions.

All framework designs in this study had a ΔE value within the clinically acceptable range (ΔE < 5.5) [ 30 - 32 ]. When comparing various framework designs, we found that crowns with dentin core framework had the highest ΔE value followed by the monolithic crowns. Simple and trestle core designs had less color change than the monolithic crowns. 

In line with our finding, a previous comparison of the color of monolithic and layered zirconia fabricated with cut-back + enamel (bi-layer), cut-back + dentin + enamel (tri-layer) approaches revealed that the monolithic crowns had higher CIE L*, a*, b*, and C* values than the layered crowns indicating the substantial impact of the veneering ceramics on the color of the zirconia restorations [ 33 ]. The study conducted by Bacchi *et al*. [ 34 ] found that bi-layer crowns demonstrated superior masking efficiency on discolored substrates compared to monolithic crowns. Rayyan *et al*. [ 33 ] compared the amount of color difference in 5 different coping designs for an upper right maxillary first molar, which were defined as full-veneer coping covering to finish line (control), 1mm cervical-shoulder (CS), monolithic zirconia with window cut-back on the buccal surface (BW), monolithic zirconia with window cut-back on buccal, lingual and mesial surfaces (3W) and circular projections of 1 mm on palatal cusps and mid-palatal surface (MM). They stated that, while there was no significant difference between the given designs, veneering aids in the reduction of color differences in monolithic zirconia. [ 33 ]. In addition to the veneering effect, it has been stated that monolithic zirconia has been found to possess greater translucency compared to framework zirconia with the same thickness [ 35 ]. According to Tuncel *et al*. [ 35 ], at the thickness of 1 mm, the average translucency parameter (TP) value was recorded as 16.4 for monolithic zirconia and 7.0 for framework zirconia. Therefore, higher values of monolithic zirconia can be attributed to the increased translucency of these crowns. Fabricating the monolithic crowns with less translucent materials can increase the masking ability of these restorations [ 33 ].

Due to the translucency of zirconia, light can pass through all zirconia crowns and interact with the underlying materials (i.e., luting cement or try in-paste). Therefore, the shade of try in-paste and cement plays a role in determining the final color of the restoration [ 36 ]. In 2009, Chang *et al*. [ 37 ] indicated that the shade of leucite glass-ceramic and zirconia crowns could cause noticeable color changes in the cervical and middle portions when particular combinations of abutment tooth material, cement, and ceramic crowns are used. In this study, the highest ΔE values were observed when the B1 shade was used and the lowest ΔE value was for the A1 shade. Regardless of the try in-paste shade, the dentin core design had the highest color difference in the incisal and middle sections, whereas the monolithic crowns showed the largest ΔE value in the cervical area. On average, the cervical area had the largest ΔE value. It appears that the effect of cement shade and tooth portion on color change is determined by the restoration thickness. Prior research has established that the thickness of the monolithic zircon directly influences its ultimate color [ 38 - 39 ]. Malkondu *et al*. [ 38 ] conducted a study to assess the alterations in color of monolithic zirconia using two different thicknesses (0.6 and 1mm) and three different types of resin luting agents. According to their findings, the average ΔE values for zirconia that is 0.6mm thick were greater than the values for zirconia that is 1mm thick. According to Tabatabian *et al*. [ 39 ], achieving a satisfactory final color requires a crown thickness of at least 0.9mm. On the other hand, Sancaktar *et al*. [ 40 ] discovered that there was no significant difference in the ΔE values of IPS emax CAD (LT C14) and Celtra Duo (LT C14) when utilized as full ceramic materials with thicknesses of 0.4 and 0.6mm when they were cemented with various backgrounds and resin cements. Dai *et al*. [ 36 ] sintered a layer of opaque porcelain onto the Co-Cr alloy substrate. They found that zirconia crowns with a thickness ranging from 1.2 to 1.5mm achieved clinically acceptable color differences (ΔE < 5.5) when used with any try-in paste shade, while restorations with a thickness of 0.7-1.0 mm in most shades achieved clinically acceptable color differences when appropriate shades of try-in paste were selected. Fachinetto *et al*. [ 41 ] conducted an in vitro investigation to assess the impact of try-in paste shade, ceramic type, and thickness on the color differences observed when cementing CAD/CAM monolithic ceramics onto discolored tooth substrates. Six different types of ceramics (high-translucent lithium disilicate (LD-HT), medium-translucent lithium disilicate (LD-MT), low-translucent lithium disilicate (LD-LT), low-translucent leucite (LC-LT), feldspathic ceramic (FC), and BL1 low-translucent lithium disilicate stained to A1 shade (LD-BL1-LT) at thicknesses of 0.5 mm, 1.0 mm, and 1.5 mm were used in this study. Their research disclosed that the color differences were influenced by all investigated factors. LD-LT and LC-LT ceramics, along with Opaque White try-in paste, generally resulted in lower ΔE00 values. The optimal ceramic thickness changed depending on the discoloration of the substrate. By using a ceramic thickness of 1.0 mm, it was feasible to achieve a ΔE00 value below the threshold of perceptibility for substrates C2 and A3, as well as a ΔE00 value below the threshold of acceptability for C3 and B3. The ΔE00 values obtained for B2, A3, and C2 were below the acceptable criterion when using a ceramic thickness of 0.5mm. The cement's masking effect can serve as an alternative for increasing the thickness of the restoration [ 36 ]. 

Try-in pastes can serve as guidance in resin luting agent selection [ 42 ]. Research suggests that try-in pastes may not match the color of resin luting agents used for all-ceramic restorations, despite their thickness [ 42 - 43 ]. A ΔE value of less than 2.0 (0.5, 0.8, or 1.0mm) indicates no perceptible color difference between resin luting agents and their try-in pastes [ 20 ]. ALGhazali *et al*. [ 44 ] found that the color difference between try-in pastes and cured resin luting agents ranged from ΔE values of 1.05 to 3.34, which is in the clinically acceptable range. However, color agreement may not always be attained, particularly with darker and opaque luting materials [ 42 ]. Try-in pastes with matching cement agent colors enable dentists, patients, and technicians to assess tooth/crown color, ensuring aesthetic expectations are met [ 20 , 29 , 44 ]. While various manufacturers may assign different names to specific shades of try-in paste, the shades we examined would likely encompass most of them. These shades are suitable for a wide range of patients. Hence, the findings of this study have the potential to offer useful guidance for selecting proper cement shades in clinical practice. However, it should be noted that color matching between the try-in paste and the accompanying resin cement is not consistently accomplished especially on the incisal and cervical tooth regions [ 21 ]. 

## Conclusion

The current investigation demonstrated that the monolithic and dentin core designs had the highest ΔE, while the simple core group had the lowest ΔE. The A2 try-in paste yielded the smallest ΔE value. These findings can provide dental laboratories with guidance on using designs that have the lowest ΔE content, such as simple core and trestle core. Similarly, dentists can choose try-in paste with the lowest ΔE content, such as A2, B1, A1, and A3, respectively.
